# Bioactive Compounds from Fruits as Preservatives

**DOI:** 10.3390/foods12020343

**Published:** 2023-01-11

**Authors:** Paulo E. S. Munekata, Mirian Pateiro, Rubén Domínguez, Gema Nieto, Manoj Kumar, Kuldeep Dhama, José M. Lorenzo

**Affiliations:** 1 Centro Tecnológico de la Carne de Galicia, Rúa Galicia n 4, Parque Tecnológico de Galicia, San Cibrao das Viñas, 32900 Ourense, Spain; 2 Department of Food Technology, Food Science and Nutrition, Faculty of Veterinary Sciences, Regional Campus of International Excellence “Campus Mare Nostrum”, University of Murcia, 30071 Espinardo, Spain; 3 Chemical and Biochemical Processing Division, ICAR—Central Institute for Research on Cotton Technology, Mumbai 400019, India; 4 Department of Biology, East Carolina University, Greenville, SC 27858, USA; 5 Division of Pathology, ICAR-Indian Veterinary Research Institute (IVRI), Bareilly 243122, India; 6 Area de Tecnoloxía dos Alimentos, Facultade de Ciencias, Universidade de Vigo, 32004 Ourense, Spain

**Keywords:** polyphenols, betalains, terpene, shelf life, safety, spoilage, meat products, fish products, dairy products, bakery products

## Abstract

The use of additives with preservative effects is a common practice in the food industry. Although their use is regulated, natural alternatives have gained more attention among researchers and professionals in the food industry in order to supply processed foods with a clean label. Fruits are essential components in a healthy diet and have also been associated with improved health status and a lower risk of developing diseases. This review aims to provide an overview of the main bioactive compounds (polyphenols, betalain, and terpenes) naturally found in fruits, their antioxidant and antimicrobial activity in vitro, and their preservative effect in different foods. Many extracts obtained from the skin (apple, grape, jabuticaba, orange, and pomegranate, for instance), pulp (such as red pitaya), and seeds (guarana, grape, and jabuticaba) of fruits are of great value due to the presence of multiple compounds (punicalagin, catechin, gallic acid, limonene, β-pinene, or γ-terpinene, for instance). In terms of antioxidant activity, some fruits that stand out are date, jabuticaba, grape, and olive, which interact with different radicals and show different mechanisms of action in vitro. Antimicrobial activity is observed for natural extracts and essential oils (especially from citrus fruits) that limit the growth of many microorganisms (*Bacillus subtilis*, *Escherichia coli*, *Penicillium digitatum*, and *Pseodomonas aeruginosa*, for instance). Studies in foods have revealed that the use of extracts or essential oils as free or encapsulated forms or incorporated into films and coatings can inhibit microbial growth, slow oxidative reactions, reduce the accumulation of degradative products, and also preserve sensory attributes, especially with films and coatings. Future studies could focus on the advances of extracts and essential oils to align their use with the development of healthier foods (especially for meat products) and explore the inhibition of spoilage microorganisms in dairy products, for instance.

## 1. Introduction

Quality decay in foods leads to modifications in safety, sensory attributes, and nutritional value [1,2]. Microbial growth, enzymatic activity, physical alterations, mechanical damage, and oxidative reactions are the main mechanisms leading to quality decay, which can also lead to alterations in the sensory properties of foods [3,4,5,6]. Food preservation involves a complex combination of factors comprising the food characteristics, processing conditions and technology, and storage conditions to retain or improve characteristics, which can be achieved with the strategic use of ingredients and processing and storage technologies [7,8,9,10].

In order to delay quality loss, additives with preservative effects are added to processed food. Although their use is regulated, the additives indicated by E-numbers (codes used to identify regulated additives in food labels) or non-familiar names have been raising concerns among consumers in the last decades, especially with regard to processed foods. In this sense, the clean-label movement has been gaining importance due to the perception of potential health risks derived from the consumption of food additives, which has in turn generated the concept of clean-label food [11]. In this sense, the use of natural extracts (rich in phenolic compounds) and essential oils (rich in terpenes) has been suggested to improve the safety and extend the shelf life of food [12,13,14,15,16].

Increasing evidence suggests that some compounds naturally present in fruits are responsible for biological effects in health [17,18,19]. This review aims to provide an overview of the main bioactive compounds found in fruits and their application (in the form of natural extracts obtained from solvent extracts and essential oils obtained from hydrodistillation) in the preservation of meat products, fish and fish products, milk and dairy products, fruits, and vegetables.

## 2. Bioactive Compounds Found in Fruits

**Figure 1:**
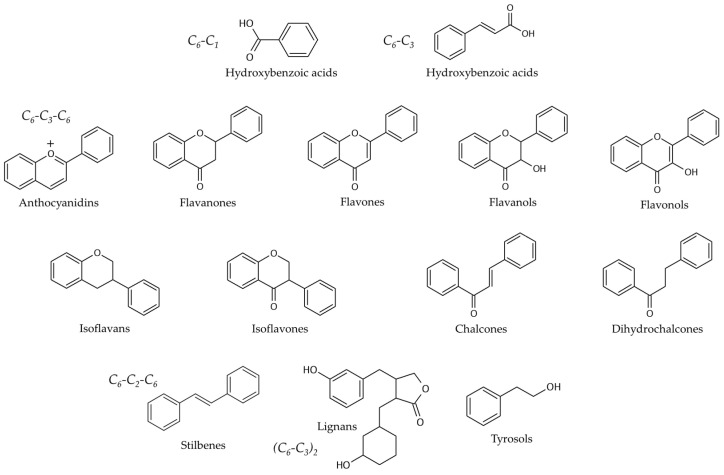
Basic chemical structures of main categories of polyphenols.

Fruits are excellent sources of bioactive compounds. Among the plethora of compounds found in these sources, some classes stand out due to their positive effects in health and also their role in the preservation of foods: polyphenols, betalains, and terpenes. Polyphenols are secondary metabolites from plant metabolism that are induced by an interaction with biotic factors (such as plant-pathogenic microorganisms and herbivorous animals) and abiotic factors (UV radiation, for instance) [20,21]. In general, this category of compounds (Figure 1) can be classified into phenolic acids (C_6_-C_1_ and C_6_-C_3_ skeleton for hydroxybenzoic and hydroxycinnamic acids, respectively), flavonoids (C_6_-C_3_-C_6_ skeleton), stilbenes (C_6_-C_2_-C_6_ skeleton), lignans (C_6_-C_3_)_2_), and other polyphenols (variable skeleton, such as tyrosol) [20].

Flavonoids are of great interest among the scientific community due to their positive effects in health, protection against the development of diseases, and their potential use to ameliorate and assist in the management of chronic diseases [22,23,24]. This class can be separated into flavonols (such as kaempferol and quercetin), flavones (such as nobiletin and tangeretin), isoflavones (such as biochanin A and formononetin), flavanones (such as hesperetin and naringenin), anthocyanidins (such as cyanidin, delphinidin, and pelargonidin), and flavanols (such as catechin, epicatechin, and procyanidins) [25,26]. Phenolic acids are a key category composed of two main groups: hydroxybenzoic acids (such as gallic acid) and hydroxycinnamic acids (caffeic acid, for instance) [25,26]. The large group of polyphenols also contains tyrosols (phenolic alcohols) [27] and dihydrochalcones [28]. Table 1 compiles phenolic compounds and some sources among fruits.

One key source of polyphenols is walnuts. Some of the main polyphenols identified in walnuts are pedunculagin/casuariin isomer, strictinin/isostrictinin isomer, trigalloyl-HHDP-glucose isomers, tellimagrandin 1 isomer, pterocarinin A isomer, castalagin/vescalagin isomers, bis-HHDP-glucose derivative, casuarin/casuarictin isomer, ellagic acid and its derivatives, cyanidin-galactoside, cyanidin-glucoside, cyanidin-arabinoside, cyanidin-pentoside, gallic acid derivative, *p*-coumaric acid hexoside derivative, tetragalloyl glucose, brevifolin, phloridzin [49]. Another source of polyphenols is apple (*Malus domestica*). In this fruit, many polyphenols have been characterized, such as quercetin, quercetin-3-glucoside, quercetin-3-*O*-galactoside, cyanidin-3-galactoside, procyanidin dimers, epicatechin, catechin belonging to the flavonoid group; phloridzin, a dihydrochalcone; and gallic acid, a hydroxybenzoic acid [31,45,46,50]. It is relevant to mention that many of these compounds are located in the peel (their presence is related to protection against UV radiation), which makes pomace a source of polyphenols [51].

Berries (cranberry, grape, and jabuticaba, for instance) are relevant sources of polyphenols, especially anthocyanidins that can also impart intense blue-to-reddish colors into these fruits [52,53]. For instance, cranberry (*Vaccinium* subg. *Oxycoccus*) contains phenolic acids (benzoic acid, *p*-coumaric acid, caffeic acid) and flavonoids (cyanidin-3-*O*-galactoside, myricetin, peonidin-3-*O*-galactoside, and quercetin) [29,30]. Grape (*Vitis vinifera*) can be indicated as one of the important sources of polyphenols among fruits. In the peels and seeds of red grape cultivars, flavonoids are the main group, which is comprised of epicatechin, catechin, delphinidin 3-*O*-glucose, malvidin 3-*O*-glucose, malvidin 3-*O*-*p*-coumaroylglucoside, peonidin 3-*O*-glucose, quercetin 3-*O*-glucoside, and quercetin 3-*O*-glucuronide [32,33,44,47]. Similarly, polyphenols can be found in *jabuticaba* (*Myrciaria cauliflora*), a berry found in tropical countries [54]. The most relevant polyphenols (anthocyanins) in this fruit are located in the peel and seeds: delphinidin-3-glucoside, cyanidin-3-*O*-glucoside, quercetin-3-*O*-rutinoside, ellagic acid, and gallic acid [34,35,36]. Pomegranate (*Punica granatum*) peel has been pointed out as a source of polyphenols. Punicalagin, ellagic acid glucoside, ferulic acid, and chyrsin have been identified in this fruit in recent studies [42,43].

The presence of bioactive compounds in watermelon (*Citrullus lanatus*) was indicated in a recent study [55]. The rind has been suggested as a relevant source of polyphenols due to the presence of phenolic acids (hydroxybenzoic and hydroxycinnamic acids) and vanillin [40]. Likewise, pequi (*Caryocar* spp.) is also a source of phenolic acids and their conjugates: gallic acid, ellagic acid, and ellagic acid deoxyhexoside [37].

Phenolic acids can also be found in date palm (*Phoenix dactylifera* L.), particularly caffeic, *p*-coumaric, protocatechuic, and ferulic acids [41]. This fruit is mainly produced in Asian and North African countries and is a source of nutrients [56]. Olive is a natural source of polyphenols that remain in the pomace from olive processing [57]. The most relevant compounds belong to the tyrosol category: oleuropein, oleuroside, hydroxytyrosol, and tyrosol [48].

Guarana (*Paullinia cupana*) is a fruit native to the Amazon rainforest located in South America [58]. The main polyphenols in the seeds of this fruit belong to the flavanol group: catechin, epicatechin, and their polymeric forms, proanthocyanidins [59,60]. Likewise, kiwifruit (*Actinidia deliciosa*), a native fruit from Asia’s temperate forests that is largely produced around world, has been indicated as a source of polyphenols [61]. The presence of phenolic acids (caffeic acid hexoside) and flavonoids (quercetin 3-*O*-rhamnoside and quercetin 3-*O*-rhamnoside) in kiwifruit was reported in a recent study [44]. It is important to note that the use of mass spectroscopy methods is recommended for the elucidation of complex polyphenol structures, accurate molecular mass determination, and reducing the risk of misidentification [62].

Betalains (Table 2) are another relevant class of natural compounds that can assist in the preservation of foods, especially as natural colorants (red and yellow pigments). These natural pigments are comprised of two classes: betacyanins and betaxanthins, which impart red-to-violet or yellow-to-orange colors, respectively [63]. These compounds can be found in red pitaya (*Hylocereus* sp.), a tropical fruit native to South America [64]. Betanin, iso-phyllocactin-I, neobetanin, phyllocactin I, and phyllocactin II have been identified in this fruit [39,65]. Moreover, these fruits also contain polyphenols (catechin, cyanidin 3,5-diglucoside, gallic acid, and ellagic acid) [38,39].

Terpenes (Table 2) are compounds naturally found in fruits. These compounds (along with other aromatic compounds) are the main components of essential oil extracts from diverse sources, which also include foods [14]. The classification of terpenes is based on the number of the isoprene structure (C_5_H_8_) in the structure of the molecule: C_10_ indicates monoterpenes, C_15_ indicates sesquiterpenes, and C_20_ indicates diterpenes [76]. Citric fruits are important sources of terpenes, especially limonene, α-pinene, β-pinene, myrcene, and γ-terpinene [66,67,68,69,70,71,72,73,74,75]. The aspects related to the antioxidant and antimicrobial activities and technological use of these compounds in different foods are described in detail in the following sections.

## 3. Antioxidant and Antimicrobial Activity of Natural Bioactive Compounds Found in Fruits

The preservative effect of these compounds can be characterized by preliminary in vitro protocols. In terms of antioxidant activity, some commonly applied methods involve the capacity to donate an electron (such as 2,2-diphenyl-1-picrylhydrazyl or 2,2′-azino-bis(3-ethylbenzothiazoline-6-sulfonic acid), also known as DPPH and ABTS, respectively) or hydrogen atom transfer (oxygen radical absorbance capacity (ORAC) and ferric-reducing ability of plasma method (FRAP), for instance) [77]. The wide use of bioactive compounds in the food science field is related to use of unsophisticated equipment, simple protocols, reduced cost per sample, and the possibility of characterizing both isolated compounds and complex mixtures (such as natural extracts from aqueous or hydroethanolic extraction and essential oils obtained from hydrodistillation) [78]. The results obtained from introductory methods provide an initial evaluation of different sources of natural bioactive compounds showing antioxidant activity for applications in foods [78]. Some examples of the characterization of antioxidant activity, as well as the total phenolic content (TPC), are displayed in Table 3.

The presence of phenolic compounds is commonly related to the antioxidant activity in different sources of extracts and essential oils. For instance, the extract of date obtained by Qureshi et al. [80] was indicated as a rich source of phenolic compounds with the capacity to scavenge radicals, particularly DPPH and ABTS. A similar inference can also be applied to jabuticaba peel [83] and red pitaya pulp [87] extracts, which can scavenge the DPPH radical and reduce capacity by the FRAP method. Another relevant source of antioxidant compounds is olive, which has been characterized in terms of ABTS, DPPH, ORAC, and FRAP methods, which suggests that the olive’s antioxidants may delay oxidative reactions by different mechanisms. This consideration is an important aspect supporting the recommendation to explore more than one method/mechanism of antioxidant activity [78]. It is important to remember that natural extracts and essential oils are complex mixtures, and their characterization supports their capacity to prevent oxidative reactions by different mechanisms [92]. Variations in terms of antioxidant activity among extracts can be explained by the natural variability of phenolic compounds to exert antioxidant activity. In other words, the structure–activity relationship [92] plays an important role in explaining, at least in part, the differences observed in Table 3.

Antimicrobial activity is another key aspect related to the bioactive compounds found in fruits. In vitro methods aim to characterize the capacity to inhibit a select microorganism. In most cases, the efforts are centered around the inhibition of a pathogenic microorganism (Table 3). The study carried out by Martínez et al. [85] observed increasing values of the inhibition radius due to the exposure of *Listeria monocytogenes* and *Staphylococcus aureus* to olive extract rich in hydroxytyrosol (dilutions using 30–90 μL of extract). Conversely, a limited effect against *Escherichia coli* was observed, which was inhibited using a low dilution (90 μL of extract). Other studies obtained results that corroborate the presence of antimicrobial compounds that can be found and extracted from pomegranate peel [86,90]. Another characterization of antimicrobial activity was reported for orange peel essential oil [72]. The effect of the concentration was observed in this study against *Bacillus subtilis*, *Candida albicans*, *Pseudomonas aeruginosa*, *Staphylococcus aureus*, and *Escherichia coli*. In other words, the inhibition of pathogenic microorganisms is an important finding to support the use of natural extracts and essential oils in improving the safety of food products.

The inhibition of spoilage microorganisms is another key aspect observed in studies of antimicrobial activity in vitro (Table 3). One relevant example is the study performed by Kharchoufi et al. [91] with pomegranate peel extract. A concentration-dependent effect of pomegranate extract in the inhibition zone of *Penicillium digitatum* (an important spoilage fungus in oranges during the post-harvest period) was observed, which indicates pomegranate extract as a potential candidate to control the growth of this fungus.

**Figure 2:**
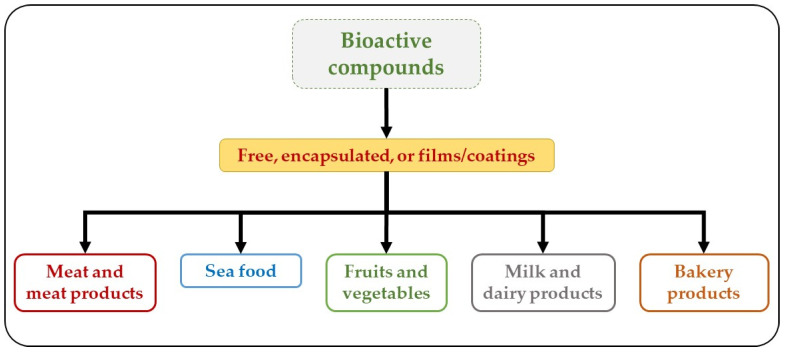
Schematic representation of the use of bioactive compounds from fruits as food preservatives.

The mechanism explaining the effect of natural bioactive compounds to inhibit microbial growth is related to multiple effects in microbial metabolism that ultimately limit growth and activity. Polyphenols and terpenes can affect microbial normal metabolism and limit the capacity to resist the damage imposed by these compounds. Recent studies exploring the mechanism of action of natural antimicrobials indicate the inhibition of microbial capacity to repair and synthesize membrane, synthesize proteins, duplicate DNA, and perform related processes [93,94,95]. The presence of many bioactive compounds in fruits supports the potential application of extracts and isolated compounds in the production of foods as preservatives (Figure 2).

## 4. Application of Fruit Bioactive Compounds in Meat and Meat Products

The incorporation of natural extracts rich in polyphenols in meat products is of great value to improve the shelf life of fresh meat and meat products (Table 4). In the case of fresh meat, the strategy consists of the preparation of a film containing the natural extract. For instance, Jiang et al. [96] obtained significant improvements in terms of color, inhibition of microbial growth, lipid oxidation, and sensory properties with a film produced with lemon essential oil. The experiment revealed that using 3% essential oil in the formulation of the film was the most effective concentration to preserve pork meat. Applying the same strategy to preserve fresh beef meat, Mehdizadeh et al. [97] explored the effect of pomegranate peel extract. In this study, the use of film containing pomegranate peel extract delayed the growth of spoilage microorganisms during refrigerated storage. The maximum allowed total microbial growth (8 log CFU/g) by the UK government for control and wrapped samples (at 0.5 and 1% in the film) was reached after 4 and 21 days, respectively. A similar effect was indicated for lipid oxidation between control and wrapped samples after 21 days of storage (≈1.5 vs. 2.5 mg malondialdehyde/kg sample for wrapped samples and controls, respectively). Moreover, the samples wrapped with the active film also received higher scores than the fresh beef in the control treatment (film without essential oil). Likewise, a recent experiment with pomegranate peel powder reported a significant reduction in microbial growth and lipid oxidation as well as improvements in the color and odor of minced beef stored at 4 °C for 15 days [42].

A similar preservative effect was reported from the incorporation of polyphenol-rich extracts in patties, fresh sausages, and cooked sausages (Table 4). A relevant example is the use of apple peel powder in a coating solution to preserve raw beef patties [98]. The samples coated with the natural extract displayed lower levels of lipid oxidation and total microbial growth in comparison to control samples. It is also relevant to mention that the growth of *Salmonella enterica* was significantly inhibited during the storage period (3.3 vs. 4.5 log CFU/g for coated and uncoated samples, respectively). A related experiment with pitaya extract indicated a similar effect in the preservation of physic-chemical, oxidative, and sensory properties of pork patties during storage at 2 °C for 18 days using 1000 mg/kg of extract [87].

The study performed by Sharma and Yadav [99] indicated that pomegranate peel powder improved the preservation of chicken by inhibiting the formation of lipid oxidation products as well as the growth of total microorganisms (Table 4). Similarly, the incorporation of guarana seed extract improved the oxidative stability in lamb burgers with chia oil emulsion during 18 days of refrigerated storage (10 vs. 6 mg malondialdehyde/kg sample after 12 days of storage for patties produced without antioxidants and 250 ppm of guarana extract, respectively) [100]. An experiment with watermelon rind extract indicated a significant reduction in the formation of lipid oxidation, microbial growth, and also in the preservation of fatty acids in pork patties [88]. This study indicated that the sensory attributes were also improved due to the use of this natural extract.

Some recent studies have explored the influence of jabuticaba peel extract in the preservation of sausages (Table 4). For instance, the inclusion of jabuticaba in hydrogelled emulsion (as a fat replacer) in beef burgers was associated with the perception of pleasant taste and the right amount of seasoning and improved oxidative stability during refrigerated storage [101]. In the case of fresh pork sausage, the microencapsulated extract of this fruit reduced the formation of lipid oxidation products (0.01, 0.02, and 0.60 mg malondialdehyde/kg sample after 15 days for 40 g/kg, 20 g/kg, and control fresh sausages, respectively) and a loss of redness, but no meaningful effect was reported against total microbial growth [83]. In another experiment using the same encapsulated microbial, no effect was observed in terms of lipid oxidation or total microbial growth inhibition in cooked pork sausage [36]. Finally, the incorporation of mango peel extract improved the lipid (0.85, 0.50, 0.39, and 0.30 mg malondialdehyde/kg sample after 10 days for 0, 2, 4, and 6% of mango peel extract, respectively) and protein oxidation (especially the formation of carbonyls; 13 vs. 8–9 nmol carbonyl/mg of protein for 0 and 2–6% mango peel extract, respectively) stability of chicken sausage during refrigerated storage [102]. However, the extract reduced the stability of the meat’s red color. Additionally, cooking loss (22.4, 20.7, 22.3, and 29.3% for 6, 4, 2, and 0% mango peel extract, respectively) was significantly reduced at the end of the storage period.

Improving oxidative stability and limiting microbial spoilage can be considered the main effects of different natural ingredients rich in polyphenols and terpenes found in fruits. Sensory properties seem to be positively affected. It is also important to consider that some effects in physicochemical properties may take place, such as reduced pH or an alteration in color parameters. Further experiments should consider the incorporation of natural extracts in combination with recent advances to improve the health-related properties of meat products (reducing NaCl and increasing omega-3 fatty acids, for instance).

## 5. Application of Fruit Bioactive Compounds in Fish and Seafood

Fish, seafood, and products derived from them can be preserved with bioactive compounds extracted from fruits, which can be incorporated into coating solutions and films or as an additive (Table 5). In terms of coating solutions, a recent experiment explored the use of grapefruit, lemon, mandarin, and orange essential oils in the preservation of fresh rainbow trout fillets [103]. The results of this experiment revealed that all essential oils reduced microbial growth, the decay of sensory attributes, lipid oxidation, and TVB-N formation during refrigerated storage. Moreover, mandarin and grapefruit display the highest antimicrobial effect among all citrus extracts. A related experiment with other citrus essential oils (ponkan, bitter, and sweet orange) indicated a similar effect in fresh bream fillets. The evaluation of fillets during storage (−1 °C during 15 days) revealed that significant reductions in the microbial growth, as well as in the formation of TVB-N, in the progression of lipid oxidation and in the loss of sensorial quality were obtained [73].

In the same line of research, Zhao et al. [33] explored the effect of grape seed extract concentration (0.3, 0.6, and 0.9% in coating solution) in the preservation of fresh tilapia fillets at 4 °C for 12 days. The most effective concentration was 0.9%, whereby both lipid oxidation and microbial growth were delayed, and a better preservation of the texture was obtained. Another relevant outcome obtained from this strategy was reported by Kim et al. [104]. In this study, the preservative effect of grapefruit seed extract in fresh shrimp was evaluated during refrigerated storage. Microbial growth, TVB-N, and melanosis were delayed in samples coated with essential oil. It is relevant to mention that the study also indicated that the coating method had a decisive impact on the preservation of shrimp, particularly treating samples with chitosan and then with alginate (containing the essential oil). Additionally, a non-significant effect was observed between control and treated samples in terms of sensory attributes.

Similarly, the use of films can improve the preservation of shrimp (Table 5). For instance, Alparslan and Baygar [72] obtained lower levels of lipid oxidation, microbial growth, and TVB-N in fresh shrimp wrapped with orange peel essential oil film than in control samples (film without essential oil). Licciardello et al. [90] obtained the same protective effect using a film with pomegranate peel extract in fresh shrimp. According to the authors, the formation of TVB-N and the growth of psychrotrophic bacteria and *Pseudomonas* spp. were reduced during the refrigerated storage.

Regarding the application of natural preservatives from fruits in minced trout fillet, a recent study indicated a significant reduction in the growth of microorganisms (Enterobacteriaceae, lactic acid bacteria, psychrotrophic bacteria, *Listeria monocytogenes*, *Pseudomonas* spp., *P. fluorescens*, and *Shewanella putrefaciens*), the formation of TVB-N, lipid oxidation, and pH when using 1 and 2% grape seed extract [32]. Moreover, sensory attributes were improved in samples with this natural extract in comparison to controls. In the case of minced shrimp meat, pomegranate peel extract powder reduced the formation of lipid oxidation products, but no effect was observed in terms of microbial growth inhibition using 5 g extract/kg [86]. The study also revealed that using a higher concentration of extract induced a pro-oxidant effect. Another experiment that supports the protective effect of fruit bioactive compounds is the study carried out by Dai et al. [105] with fish mince. The incorporation of blueberry pomace and wince pomace extracts reduced the formation of oxidation products from lipid and proteins during refrigerated storage. This study also revealed that the source and the concentration of extract were both important. Higher concentrations (2 vs. 1 g/kg) of extract reduced lipid and protein oxidation, and samples treated with blueberry wine pomace displayed lower oxidation indexes.

The preservative effect of fruit extract was also observed in fish and seafood products (Table 5). For instance, the experiment carried out by Martínez-Zamora et al. [106] indicated a significant reduction in the generation of lipid oxidation products, volatile compounds, and redness using *Citrus sinensis* and pomegranate extracts in fish patties. However, the *Citrus sinensis* extract caused a significant increase in the growth of the total number of microorganisms in the fish patties, whereas no effect was reported for pomegranate extract. A related experiment with fish patties indicated a significant reduction in the formation of volatile compounds derived from lipid oxidation due to olive fruit extract [85]. However, no significant effect was observed in terms of microbial growth inhibition.

In the case of fish balls, the use of grape seed extract (in combination with sage and oregano leaf extract) reduced lipid oxidation, the TVB-N, and microbial growth for 15 days at 4 °C [107]. Finally, a recent study with grape seed extract in cooked shrimp indicated the inhibition of *Listeria monocytogenes* growth during refrigerated storage [108].

Fish and seafood products are known to have a fast deterioration process, but the process can be delayed using natural preservatives from fruits. Essential oils seem to stand out in relation to extracts rich in polyphenols, especially for microbial growth inhibition.

## 6. Application of Fruit Bioactive Compounds in Milk and Dairy Products

The preservative compounds found in fruits have also been used in milk and dairy products to prevent quality decay during storage (Table 6). Regarding the preservation of milk, Min et al. [89] observed that the peel extract of *Citrus unshiu* caused a fast reduction in *Listeria monocytogenes* counts in cow milk (whole, low fat, and skim) during refrigeration. Moreover, a concentration-dependent effect was observed for this extract when using 40 g/kg. In another experiment with cow milk, Di Maio et al. [84] evaluated the influence of nanoemulsified jabuticaba peel extract (15%) during refrigerated storage but did not observe significant changes in the pH of treated milk samples.

In the case of yogurt, a recent study indicated that the addition of apple peel extract induced changes in the properties (total solids, viscosity, and WHC) of this product that remained throughout the storage period [79]. Moreover, no significant differences in terms of pH, acidity, and LAB growth were reported. In a related experiment with yogurt, the addition of grape pomace powder was associated with changes in the acidity and redness [81]. However, the acceptability of the supplemented yogurts displayed lower scores in comparison to the control treatment. A related experiment with yogurt indicated banana peel as source of bioactive compounds to prevent lipid oxidation [105]. This study also revealed that banana peel extract did not affect the other important parameters for yogurt, pH, and syneresis.

The preservation of cheese was also reported in literature (Table 6). For instance, Khalifa et al. [109] studied the influence of cranberry fruit extract powder in white soft cheese and observed that the extract improved the appearance and total score in terms of sensory analysis. Additionally, this extract also had a concentration-dependent effect in the inhibition of lipid oxidation, microbial growth, and release of fatty acids, wherein the lowest values in these indicators were obtained from treatments produced with the highest extract concentration (1000 mg/kg). Similarly, the use of pomegranate peel extract caused a significant increase in sensory scores (appearance, aroma, bitterness, flavor, and overall acceptability) of treated Himalayan cheese after 10 days of storage [110]. This extract also reduced the progression of oxidative reactions in both lipids and proteins as well as the microbial growth of spoilage microorganisms. In the case of Paneer (a traditional Indian cheese), the inclusion of date extract increased the redness of the product, but no significant effect was observed for microbial growth or sensory attributes in comparison to control cheese [80].

An experiment was carried out by Moreira et al. [111] with *pequi* waste extract in goat *Minas Frescal* cheese. Three different strategies with this natural extract were tested: addition into the milk, incorporation into the cheese mass, and in a coating solution. The results of the experiment revealed that the incorporation of *pequi* waste extract into the milk was the most adequate strategy due to the improved texture properties and microbial quality. However, the extract caused a significant reduction in the luminosity of the cheese, regardless of strategy. A similar effect in the color of the cheese was also reported by Saito et al. [112], who added jabuticaba peel extract in petit-suisse cheese. A concentration-dependent effect in color was observed using this natural extract (15–30 g/kg), but no significant effect was obtained among treatment for sensory attributes.

Natural preservatives from fruits can assist in the preservation of milk and dairy products, especially in the prevention of an oxidative reaction using extracts rich in polyphenols. However, natural extracts and essential oils seem to have limited abilities against spoilage microorganisms during shelf life. A possible interaction with dairy components seems to explain this outcome, but additional studies are necessary to clarify this relationship.

## 7. Application of Fruit Bioactive Compounds in Fruits and Vegetables

The use of polyphenol-rich extracts and essential oils has been tested to improve the preservation of fruits and vegetables. It is important to mention that these bioactive compounds have been incorporated into coating, films, or dipping solutions in order to apply treatments in this category of foods. The use of bioactive compounds from fruits in the preservations of foods of vegetable origin is shown in Table 7. A relevant example of the preservative effect of polyphenol-rich extract in the preservation of fruit was reported by Akbari et al. [82] with pomegranate byproduct, juice, and extract, as well as kiwifruit and grape juice in dipping solution. Treating fresh-cut pear with combinations of kiwifruit juice with either pomegranate or grape juice enhanced the content of vitamin C, a* values, and sensory attributes, and it reduced peroxidase activity during storage. It is important to highlight that the increase in vitamin C in treated pears was a consequence of dipping the sample in the juice, rather than an actual reaction leading to the synthesis of vitamin C. However, the samples treated with the polyphenol-rich extracts and juices reduced L* values and firmness at the end of storage period. A similar outcome was reported by Fan et al. [31], who sprayed apple polyphenols on fresh-cut red pitaya. In this study, the treated samples displayed higher values for color, texture, TPC, and AA than controls (samples sprayed with water) after 4 days at 20 °C. Moreover, a reduction in the microbial growth and a reduction in betacyanin content were observed in this experiment.

Another relevant outcome was reported by Yousuf et al. [113] with lemon extract. This polyphenol-rich extract caused a reduction in the weight loss of fresh-cut melon, inhibited the growth of spoilage microorganisms, and diminished the loss of vitamins and sensory attributes, especially using up to 10% in coating solution. A related experiment carried out with strawberries showed that pomegranate peel extract (in a dipping solution) slowed the growth of *Botrytis cinerea* during storage [114]. Similarly, the growth of *Penicillium digitatum* in oranges was delayed by immersing them in a solution with the extract obtained from pomegranate peel [91]. Conversely, Tomadoni et al. [115] did not report a significant effect in the sensory attributes and microbial growth of fresh-cut strawberries wrapped with film enriched with pomegranate extract.

In the case of essential oils, many studies indicate that this kind of compound displays the potential to preserve fruits and vegetables (Table 7). For instance, the experiment performed by Gomes et al. [116] indicated that the preservation of red raspberries could be improved using coatings with lemon and orange essential oil. The main outcomes associated with these samples with coating treatments were the reduction in microbial growth (aerobic mesophilic bacteria, yeasts, and molds) and increased antioxidant activity in the berries. However, an increase in the weight loss of raspberries coated with lemon and orange essential oil was observed, and no significant effect was observed for sensory attributes.

Radi et al. [117] explored the influence of emulsion preparation (microemulsion vs. nanoemulsion) in the preservation of fresh-cut orange. The use of emulsified orange peel essential oil increased the pH, the scores in the sensory analysis evaluation, the content of vitamin C, and the L* values at the end of storage in comparison to untreated samples. Microbial growth inhibition was also observed in samples treated with nanoemulsion containing orange peel essential oil. Conversely, Perdones et al. [118] observed a decay in respiration rate (for both O_2_ and CO_2_), but a significant reduction in texture and sensory attributes during storage were also reported. No meaningful difference was indicated for pH, acidity, and luminosity between coated and non-coated strawberries. It is also relevant to mention that the lycopene was not an active coating component to preserve fresh–cut apples [120]. Although low a browning index was obtained with samples coated with lycopene-enriched coats, significant reductions in TPC and AA were also observed in treated samples. Additionally, no significant effect was observed in terms of microbial growth inhibition.

In the case of vegetables (Table 7), the inhibition of *Listeria monocytogenes* growth in broccoli florets was obtained by coating them with lemon and mandarin essential oil combined with non-thermal decontamination technologies (particularly with ozone, UV-C, and γ irradiation) [66]. Another experiment using the same approach (mandarin essential oils with ultraviolet-C and γ irradiation) indicated the same outcome against *Listeria monocytogenes* in green beans, but reductions in the color and firmness were also reported [119].

The use of essential oils seems to show a consistent antimicrobial activity against both pathogenic and spoilage microorganisms in fruits and vegetables. The use of essential oils in films and coatings is indicated by many studies as the main solution, but attention is still necessary to improve the preservation of other characteristics, especially texture, which may receive more attention in future experiments.

## 8. Application of Fruit Bioactive Compounds in Bakery Products

The preservation of bakery products is another relevant effect associated with bioactive compounds naturally found in fruits. An example of this preservative effect is the study carried out by Sharayei et al. [121] with pomegranate peel extract in cupcakes. The authors tested the effect of encapsulation and concentration (only for encapsulated extract). A significant reduction in the growth of molds and yeast on samples containing the natural extracts in comparison to the control treatment (without preservatives) was observed. The lowest growth rate was obtained using the encapsulated extract at 1.5% (0.12 log CFU/g), followed by 1.0% encapsulated (0.52 log CFU/g) and 0.3% free extract (1.2 log CFU/g). Additionally, both encapsulated extracts increased the overall acceptance of cupcakes in relation to control samples. A related experiment with cake indicated a similar preservative effect against yeast and molds with free and encapsulated orange essential oil [122]. Samples produced with either free or encapsulated extracts displayed 3 log CFU/g, whereas the control samples (without additives) had 4 log CFU/g after 150 days at 20 °C. A similar result was reported with Ukom cakes produced with grapefruit peel powder (2.5, 3.7, and 5 g) [123]. Cakes produced with grapefruit powder had lower peroxide values, yeast and mold counts, and it inhibited the activity of lipase (responsible for the release of fatty acids and reduced lipid oxidative stability). Moreover, sensory analysis revealed that this natural extract preserved the sensory properties of cake (appearance, taste, texture, aroma, and general acceptability) after 7 days of storage.

The incorporation of fruit bioactive compounds in films can also improve the microbial stability of bakery products. This outcome was reported in a recent study with a combination of pomegranate and orange peel extracts in films to preserve white bread for 5 days at 30 °C [124]. The samples wrapped in the bioactive film displayed lower microbial counts at the end of the storage period than the samples without packaging or packaged with polythene. Another relevant outcome from this study was the reduction in moisture loss during storage, whereby the bread packaged with bioactive packaging had lower moisture losses than unwrapped and polythene-wrapped bread. In general, natural extracts can inhibit the growth of molds and yeast in bakery products, but the effect is limited to less than 2 log CFU/g. In this sense, it seems reasonable to indicate that films can be seen as the main option to improve shelf life by inhibiting yeast and mold growth during shelf life. Additional benefits from this strategy consist of the preservation of physicochemical properties and sensory attributes.

## 9. Advantages and Disadvantages

The preservation of food against oxidative reactions and microbial spoilage, as indicated in previous sections, is a key and direct benefit that supports the continuous search for natural preservatives from fruits. Another key aspect is the alignment of food production with current trends of preference and growing preference for natural and clean-label foods [125,126]. Sources are available worldwide and from largely consumed fruits such as orange, grape, watermelon, mandarin, and pomegranate. Moreover, the production of natural ingredients is also in agreement with the development of sustainable actions and a circular economy to produce high-added-value raw materials for the food industry [127,128].

The research on natural preservatives from fruits also has important challenges for the progression towards their potential use in food products. A key aspect to consider is the difference in results between in vitro assays and experiments with food systems. The remarkable results reported from the chemical method using radicals for antioxidant activity and culture media with minimal interference may not be observed in food systems. The interaction or dilution of bioactive compounds with food components may limit their effect on oxidative reactions and spoilage microorganisms [2]. Consequently, an increase in their concentration may be necessary, which may change the odor and/or flavor of foods. Particularly for polyphenols, these compounds are perceived as bitter or astringent, which may impose an additional challenge for the development of foods using natural preservatives rich in polyphenols. The main reason for such an issue is the human perception associated with bitterness or astringency, which reflects an instinctive protection against potentially harmful compounds in foods [129]. This aspect has been pointed out in studies with children and teenagers, who generally dislike foods with astringency and bitterness as the main sensory descriptors [130]. Therefore, it seems reasonable to consider bitterness and astringency as descriptors for the sensory analysis of food with added natural preservatives rich in polyphenols and to check for the relationship of these qualities with overall acceptance during product development.

Although not discussed in the present review, the obtainment of natural extracts and essential oils is a complex task that involves the proper use of technologies and strategies to preserve the antioxidant and antimicrobial potentials of compounds naturally found in fruits. The extraction of active compounds implies the use of a solvent, of equipment and energy consumption, and the potential generation of residues. Current options are limited for extraction using food-grade solvents (mainly water or hydroethanolic solutions). Consequently, the cost of the food may be increased, depending on the technology and strategy used to obtain its natural preservative(s) [131].

## 10. Conclusions

Fruits are sources of relevant bioactive compounds with great potential applications in the preservation of foods. Extracts (rich in polyphenols) and essential oils (rich in terpenes) can effectively delay quality decay in different food products (meat and meat products, fish and seafood, milk and dairy products, fruits and vegetables), which supports their versatility and potential application as food preservatives. Date, jabuticaba, grape, citrus fruits, and olive are among the main sources that contain polyphenols, such as punicalagin, catechin, and gallic acid, and terpenes, such as limonene, β-pinene, or γ-terpinene.

The use of different strategies (free or encapsulated form of the compound, incorporation into film and coatings) is also possible and supports advances for further applications of natural preservatives at larger scales. Inhibiting microbial growth (spoilage and pathogenic microorganisms), slowing the progression of lipid oxidation, decelerating the accumulation of volatile basic nitrogen, and preserving sensory attributes are the most common outcomes derived from the use of fruit bioactive compounds. Additional studies should consider the specific needs for each food category in order to solve current specific challenges to prevent secondary effects in terms of physicochemical and sensory changes, for instance. It is also relevant to take into account the possible impact on sensory properties (especially the intensification of astringency and bitterness, as well as changes in color, odor, and flavor) that may affect consumer acceptance and align future studies with emerging trends in food production, especially health-related reformulations.

## Figures and Tables

**Table 1:** Polyphenols found in fruits.

Compound Category	Compounds	Source	Ref.
Phenolic acids			
Hydroxybenzoic acids	Benzoic acid	Cranberry fruit	[29,30]
Hydroxybenzoic acids	Gallic acid	Apple	[31]
Hydroxybenzoic acids	Gallic acid	Grape seed	[32,33]
Hydroxybenzoic acids	Gallic acid	Jabuticaba peel and seeds	[34,35,36]
Hydroxybenzoic acids	Gallic acid	Pequi pulp	[37]
Hydroxybenzoic acids	Gallic acid	Red pitaya pulp	[38,39]
Hydroxybenzoic acids	Hydroxybenzoic acid	Watermelon rind	[40]
Hydroxybenzoic acids	Protocatechuic acid	Date palm	[41]
Hydroxybenzoic acids	Ellagic acid	Jabuticaba peel and seeds	[34,35,36]
Hydroxybenzoic acids	Ellagic acid	Pequi pulp	[37]
Hydroxybenzoic acids	Ellagic acid deoxyhexoside	Pequi pulp	[37]
Hydroxybenzoic acids	Ellagic acid glucoside	Pomegranate peel	[42,43]
Hydroxybenzoic acids	Ellagic acid	Red pitaya pulp	[38,39]
Hydroxybenzoic acids/Ellagitannins	Punicalagin	Pomegranate peel	[42,43]
Hydroxybenzaldehydes	Vanillin	Watermelon rind	[40]
Hydroxycinnamic acids	Caffeic acid	Cranberry fruit	[29,30]
Hydroxycinnamic acids	Caffeic acid	Date palm	[41]
Hydroxycinnamic acids	Caffeic acid hexoside	Kiwifruit pomace	[44]
Hydroxycinnamic acids	Chlorogenic acid	Apple	[31]
Hydroxycinnamic acids	Chlorogenic acid	Apple peel	[45,46]
Hydroxycinnamic acids	Chlorogenic acid	Watermelon rind	[40]
Hydroxycinnamic acids	Ferulic acid	Date palm	[41]
Hydroxycinnamic acids	Ferulic acid	Pomegranate peel	[42,43]
Hydroxycinnamic acids	Hydroxycinnamic acid	Watermelon rind	[40]
Hydroxycinnamic acids	*p*-coumaric acid	Apple	[31]
Hydroxycinnamic acids	*p*-coumaric acid	Apple peel	[45,46]
Hydroxycinnamic acids	*p*-coumaric acid	Cranberry fruit	[29,30]
Hydroxycinnamic acids	*p*-coumaric acid	Date palm	[41]
Hydroxycinnamic acids	Sinapinic acid	Watermelon rind	[40]
Flavonoids			
Anthocyanins	Cyanidin 3,5-diglucoside	Red pitaya pulp	[38,39]
Anthocyanins	Cyanidin 3-*O*-galactoside	Cranberry fruit	[29,30]
Anthocyanins	Cyanidin 3-*O*-glucoside	Jabuticaba peel and seeds	[34,35,36]
Anthocyanins	Delphinidin 3-*O*-glucoside	Grape pomace (peel and seed)	[44,47]
Anthocyanins	Delphinidin 3-*O*-glucoside	Jabuticaba peel and seeds	[34,35,36]
Anthocyanins	Malvidin 3-*O*-glucose	Grape pomace (peel and seed)	[44,47]
Anthocyanins	Malvidin 3-*O*-*p*-coumaroylglucoside	Grape pomace (peel and seed)	[44,47]
Anthocyanins	Peonidin 3-*O*-glucoside	Grape pomace (peel and seed)	[44,47]
Anthocyanins	Peonidin 3-*O*-glucoside	Grape pomace (peel and seed)	[44,47]
Anthocyanins	Peonidin 3-*O*-galactoside	Cranberry fruit	[29,30]
Anthocyanins	Petunidin 3-*O*-glucose	Grape pomace (peel and seed)	[44,47]
Flavones	Chyrsin	Pomegranate peel	[42,43]
Flavonols	Myricetin	Cranberry fruit	[29,30]
Flavonols	Quercetin	Apple peel	[45,46]
Flavonols	Quercetin	Cranberry fruit	[29,30]
Flavonols	Quercetin 3-*O*-glucoside	Grape pomace (peel and seed)	[44,47]
Flavonols	Quercetin 3-*O*-glucuronide	Grape pomace (peel and seed)	[44,47]
Flavonols	Quercetin 3-*O*-rhamnoside	Kiwifruit pomace	[44]
Flavonols	Quercetin 3-*O*-rhamnoside	Kiwifruit pomace	[44]
Flavonols	Quercetin 3-glucoside	Apple peel	[45,46]
Flavonols	Quercetin 3-*O*-rutinoside	Jabuticaba peel and seeds	[34,35,36]
Flavonols	Rutin	Apple peel	[45,46]
Dihydrochalcones	Phloretin	Apple	[31]
Dihydrochalcones	Phloridzin	Apple peel	[45,46]
Dihydrochalcones	Phlorizin	Apple	[31]
Other polyphenols			
Tyrosols	Hydroxytyrosol	Olive pulp	[48]
Tyrosols	Oleuropein	Olive pulp	[48]
Tyrosols	Oleuroside	Olive pulp	[48]
Tyrosols	Tyrosol	Olive pulp	[48]

**Table 2:** Betalains and terpenes found in some fruits.

Source	Compound Category	Compounds	Ref.
Red pitaya pulp	Betalains	Betanin, phyllocactin I, phyllocactin-II, neobetanin, and iso-phyllocactin-I	[39,65]
Lemon	Terpenes	Limonene, β-pinene, and γ-terpinene	[66,67,68,69]
Lime	Terpenes	Limonene, γ-terpinene and β-pinene	[70,71]
Mandarin	Terpenes	Limonene, γ-terpinene, and α-pinene	[66,69]
Orange	Terpenes	Limonene and myrcene	[69,72]
Orange peel	Terpenes	Limonene and γ-terpinene	[73]
Grapefruit	Terpenes	Limonene	[69,74,75]

**Table 3:** In vitro evaluation of total phenolic content and antioxidant and antimicrobial activities.

Source	Method and Activity	Ref.
Phenolic content and antioxidant activity		
Apple peel extract	TPC: 1.39–3.30 g GAE/100 g dry weight TFC: 1.15–2.04 g QE/100 g	[79]
Date extract	TPC: 2.74–3.60 µg GAE/mL TFC: 4.77–9.06 µg CE/mL DPPH: 720–1.120 µmol Trolox/mL ABTS: 621–774 µmol Trolox/mL	[80]
Grape pomace	TPC: 20–35.0 mg GAE/g dry weight	[81]
Grape extract	TPC: 10.99 mg GAE/100 mL DPPH: 10.17% inhibition	[82]
Jabuticaba peel extract	TPC: 13.22 mg GAE/mL FRAP: 5.76 μmol Trolox/L DPPH: 36.03 mmol Trolox/L	[83]
Jabuticaba peel extract	TPC: 18.51 mg GAE/mL DPPH: 36.75 µg TEAC/mL	[84]
Kiwifruit extract	TPC: 21.32 mg GAE/100 mL DPPH: 19.84% inhibition	[82]
Olive extract	TPC: 41.4 mg GAE/g ABTS: 79.6% inhibition DPPH: 78.0% inhibition ORAC: 140.5 µM Trolox FRAP: 71.2 µM Trolox	[85]
Pomegranate peel powder	TPC: 215.2 mg/g TFC: 70.4 mg/g DPPH (EC_50_): 17.8 μg/mL	[42]
Pomegranate peel extract powder	TPC: 101–169 mg GAE/100 g	[86]
Pomegranate extract	TPC: 33.15 mg GAE/100 mL DPPH: 20.34% inhibition	[82]
Pomegranate byproduct extract	TPC: 127.51 mg GAE/100 mL DPPH: 93.61% inhibition	[82]
Red pitaya pulp extract	TPC: 268.13 mg of GAE/100 g DPPH (EC_50_): 229 mg Trolox/100 g FRAP: 825.40 μmol Fe^+2^/100 g.	[87]
Watermelon rind extract	DPPH: 77.46% inhibition ABTS: 75.57% inhibition FRAP: 77.5 mM Fe^2+^/mL SASA: 47.5% inhibition	[88]
Antimicrobial activity		
Citrus unshiu peel extract	*Bacillus cereus* (MIC): 10–20 mg/mL *Staphylococcus aureus* (MIC): 10–20 mg/mL *Listeria monocytogenes* (MIC): 10–20 mg/mL	[89]
Olive extract	*Escherichia coli* (IZ): 11 mm *Listeria monocytogenes* (IZ): 6–13 mm *Staphylococcus aureus* (IZ): 8–25 mm	[85]
Orange peel essential oil	*Bacillus subtilis* (IZ): 7.0–7.5 mm *Candida albicans* (IZ): 10–12 mm *Pseodomonas aeruginosa* (IZ): 10.5–13 mm *Stapylococcus aureus* (IZ): 8.5–12.5 mm *Escherichia coli* (IZ): 9.5–12.0 mm	[72]
Pomegranate peel extract	*Pseudomonas putida* (IZ): 3–8 mm	[90]
Pomegranate peel extract powder	*Bacillus cereus* (IZ): 13–19 mm *Escherichia coli* (IZ): 12–17 mm *Pseudomonas aeruginosa* (IZ): 12–16 mm *Staphylococcus aureus* (IZ): 13–19 mm	[86]
Pomegranate peel extract	*Penicillium digitatum* (IZ): 0.3–4.1 mm	[91]

ABTS: 2,2′-azino-bis(3-ethylbenzothiazoline-6-sulfonic acid); CE: catechin equivalent; DPPH: 2,2-diphenyl-1-picrylhydrazyl; EC_50_: extract concentration decreasing the initial DPPH radical concentration by 50%; FRAP: ferric-reducing ability of plasma; GAE: gallic acid equivalent; IZ: inhibition zone; MIC: minimum inhibitory concentration; ORAC: oxygen radical absorbance capacity; QE: quercetin Equivalent; SASA: superoxide anionic scavenging activity; TFC: total flavonoid content; and TPC: total phenolic content.

**Table 4:** Preservative effect of fruit bioactive compounds in meat and meat products.

Source	Meat and Meat Products (Extract Concentration)	Storage Conditions	Results	Ref.
Lemon essential oil	Fresh pork meat (1–4% in film)	21 days at 4 °C	↑ Color, odor, and overall acceptance (SA) ↓ MG and LO	[96]
Pomegranate peel extract	Fresh beef (0.5 and 1% in film)	21 days at 4 °C	↑ Overall acceptance ↓ MG and LO No effect: color and odor (SA)	[97]
Pomegranate peel powder	Minced beef meat (1 and 1.5%)	15 days at 4 °C	↑ Color and odor (SA) ↓ LO, TVB-N, WHC, CL, pH, and MG	[42]
Apple peel powder	Raw beef patties (3% in coating solution)	10 days at 4 °C	↓ LO, MG, and *Salmonella enterica* counts	[98]
Red pitaya pulp extract	Raw pork patties (250, 500 and 1000 mg/kg)	18 days at 2 °C	↑ PAA, a*, and color (SA) ↓ pH, L*, b*, LO, and PO No effect: texture, CL, and SA	[87]
Pomegranate peel powder	Raw chicken patties (powder and extract: 2 and 9 g/100 g, respectively)	16 days for 4 °C	↑ Texture ↓ L*, LO, and MG No effect: a*, b*, and pH	[99]
Guarana seed extract	Raw lamb burgers with chia oil emulsion (250 mg/kg)	18 days at 2 °C	↑ PAA, pH, a*, color (SA) ↓ L*, b*, metMb, LO, PO, and VC	[100]
Watermelon rind extract	Cooked pork patties (1000 mg/kg)	28 days at 4 °C	↑ Flavor, juiciness, tenderness, and overall acceptance (SA) ↓ MG, LO, and FFA No effect: pH	[88]
Jabuticaba peel extract	Raw and cooked beef burgers (6, 8, and 10% in hydrogelled emulsion)	120 days at −18 °C	↑ Pleasant taste and seasoning in the right amount (SA) ↓ LO	[101]
Jabuticaba peel extract	Fresh pork sausage (20 and 40 g/kg)	15 days 1 °C	↑ a* ↓ LO, pH, L*, b*, and color (SA) No effect: MG	[83]
Jabuticaba peel extract	Cooked pork sausage (20 g/kg)	56 days at 4 °C	↑ Texture and flavor (SA) ↓ pH, a*, b*, and color (SA), No effect: LO, MG, and overall acceptance (SA)	[36]
Mango peel extract	Cooked chicken sausage (2, 4, and 6%)	10 days at 4 °C	↓ pH, L*, a*, b*, CL, LO, and PO	[102]

a*: Color from green to red; b*: color from blue to yellow; CL: cooking loss; FFA: free fatty acids; L*: luminosity; LO: lipid oxidation; MetMb: metmyoglobin; MG: microbiological growth; PAA: product antioxidant activity; PO: protein oxidation; SA: sensory attributes; TVB-N: total volatile basic nitrogen; VC: volatile compounds; and WHC: water-holding capacity.

**Table 5:** Preservative effect of fruit bioactive compounds in fish, seafood, and derived products.

Source	Fish, Seafood, and Derived Products (Extract Concentration)	Storage Conditions	Results	Ref.
Grapefruit, lemon, mandarin, and orange essential oil	Fresh rainbow trout fillets (4% in coating solution)	15 days at 4 °C	↑ SA ↓ TVB-N, pH, LO, FFA, and MG	[103]
Ponkan, bitter, and sweet orange essential oils	Fresh bream fillets (4 mL/L in coating solution)	15 days at −1 °C	↑ SA and texture ↓ TVB-N, pH, LO, and MG	[73]
Grape seed extract	Fresh tilapia fillets (0.3, 0.6, and 0.9% in coating solution)	12 days at 4 °C	↑ Texture ↓ MG and PO	[33]
Grapefruit seed extract	Fresh shrimps (2% in coating solution)	15 days at 4 °C	↓ MG, TVB-N (coating method), and melanosis No effect: SA	[104]
Orange peel essential oil	Fresh shrimp (2% in film)	15 days at 4 °C	↓ MG, LO, and TVB-N	[72]
Pomegranate peel extract	Fresh shrimp (0.361 g/mL film solution)	6 days at 4 °C	↓ MG and TVB-N No effect: color (SA)	[90]
Grape seed extract	Minced trout fillet (1 and 2%)	11 days at 4 °C	↑ SA ↓ MG, TVB-N, LO, and pH	[32]
Pomegranate peel extract powder	Minced shrimp (5–20 g/kg)	28 days at 4 °C	↓ LO No effect: MG	[86]
Blueberry pomace extract	Fish mince (1 and 2 g/kg)	14 days at 4 °C	↓ LO and PO	[105]
Blueberry wine pomace extract	Fish mince (1 and 2 g/kg)	14 days at 4 °C	↓ LO and PO	[105]
*Citrus sinensis* and pomegranate extract	Fish patties (200 mg/kg)	11 days at 4 °C	↑ L*, b*, and MG (*Citrus sinensis*) ↓ a*, LO, and VC No effect: PO, trimethylamine content, and TVB-N	[106]
Olive extract	Fish patties (200 mg/kg)	11 days at 4 °C	↓ VC No effect: MG	[85]
Grape seed extract	Fish ball (0.01% with 0.1% of sage extract and 0.1% oregano extract)	15 days at 4 °C	↓ LO, pH, TVB-N, and MG	[107]
Grape seed extract	Cooked shrimp (1%)	12 days at 4 °C	↓ *Listeria monocytogenes* growth No effect: color	[108]

a*: Color from green to red; b*: color from blue to yellow; FFA: free fatty acids; L*: luminosity; LO: lipid oxidation; MG: microbiological growth; PO: protein oxidation; SA: sensory attributes; TVB-N: total volatile basic nitrogen; and VC: volatile compounds.

**Table 6:** Preservative effect of fruit bioactive compounds in milk and dairy products.

Source	Milk and Dairy Products (Extract Concentration)	Storage Conditions	Results	Ref.
*Citrus unshiu* peel extract	Cow milk (10–40 g/kg)	21 days at 4 °C	↓ *Listeria monocytogenes* counts	[89]
Jabuticaba peel extract	Cow milk (15%)	60 days at 8 °C	No effect: pH	[84]
Apple peel extract	Yogurt (10–50 g/kg)	21 days at 4 °C	↑ Total solids, WHC, viscosity, and SA No effect: pH, acidity, and MG	[79]
Grape pomace	Yogurt (10, 30 and 50 g/kg)	21 days at 4 °C	↑ Acidity and a* ↓ pH, L*, b*, and SA	[81]
Banana peel extract	Yogurt (1000 μL/100 g)	28 days at 4 °C	↓ LO No effect: pH and syneresis	[105]
Cranberry fruit extract powder	White soft cheese (500, 750, and 1000 mg/kg)	8 weeks at 2 °C	↑ Appearance and total score (SA) ↓ Acidity, FFA, LO, and MG	[109]
Pomegranate peel extract	Himalayan cheese/ kalari (25, 50, and 75 mg/mL film forming solution)	30 days at RgT	↑ SA ↓ MG, LO, and PO	[110]
Date extract	Paneer (20 g/100 g)	8 days at 5 °C	↓ pH No effect: MG and SA	[80]
*Pequi* waste extract	Goat *Minas Frescal* cheese (6.25 mL/L in milk, mass, or coating solution)	21 days at 4 °C	↑ a* ↓ LAB, *Lactococcus* spp., and L*	[111]
Jabuticaba peel extract	Petit-suisse cheese (15–30 g/kg)	28 days at 8 °C	↑ a* ↓ pH, L*, and b* No effect: SA	[112]

a*: Color from green to red; b*: color from blue to yellow; FFA: free fatty acids; L*: luminosity; LO: lipid oxidation; MG: microbiological growth; PO: protein oxidation; RgT: refrigerated temperature; SA: sensory attributes; and WHC: water-holding capacity.

**Table 7:** Preservative effect of fruit bioactive compounds in fruits and vegetables.

Source	Fruits and Vegetables (Extract Concentration)	Storage Conditions	Results	Ref.
Pomegranate byproduct, juice, and extract; kiwifruit and grape juice and extract	Fresh-cut pear (0.3% in coating solution; individually or combined)	7 days at 4 °C	↑ Ascorbic acid, a*, and SA ↓ Firmness, L*, and peroxidase activity No effect: polyphenol oxidase	[82]
Apple polyphenols	Fresh-cut red pitaya (5 g/L sprayed solution)	4 days at 20 °C	↑ Color, texture, TPC, and AA ↓ Betacyanin and MG	[31]
Lemon extract	Fresh-cut melon (5, 10 and 15% in coating solution)	12 days at 4 °C	↓ Weight loss, MG, vitamin C loss, and pH No effect: L* and SA	[113]
Pomegranate peel extract	Strawberries (0.75, 1.5 and 3.0% in solution)	2 days at 0 °C followed by 3 days at 20 °C	↓ *Botrytis cinerea* growth	[114]
Pomegranate peel extract	Oranges (0.361 g/mL in coating solution)	6 days at 25 °C	↓ *Penicillium digitatum* growth	[91]
Pomegranate extract	Fresh-cut strawberries (360 and 720 μg/mL in film solution)	7 days at 5 °C	No effect: MG and SA	[115]
Lemon, lime, and orange essential oil	Red raspberries (0.1 and 0.2% in coating solution)	15 days at 4 °C	↑ L*, texture, weight loss (lemon and orange), TPC (lime), and AA ↓ MG (lemon and orange) No effect: SA	[116]
Orange peel essential oil	Fresh-cut orange (0.5 and 1% in coating solution)	17 days at 4 °C	↑ SA, pH, and TA decay ↓ Ascorbic acid loss, weight loss, L* reduction, and MG (nanoemulsion)	[117]
Lemon essential oil	Strawberry (3% in coating solution)	14 days at 5 °C	↓ Texture, RR O_2_ and CO_2_, and SA No effect: pH, TA, and L*	[118]
Lemon and mandarin essential oils	Broccoli florets (0.05% in coating solution)	13 days at 4 °C	↓ *Listeria monocytogenes* growth	[66]
Mandarin essential oils	Green beans (0.05% in coating solution)	15 days at 4 °C	↓ *Listeria monocytogenes* growth, loss of firmness, and color changes	[119]
Lycopene from tomato byproducts	Fresh-cut apple (0.5, 1, and 2 g/L in coating solution)	9 days at 5 °C	↓ Browning index, TA, TPC, and AA No effect: pH and MG	[120]

a*: Color from green to red; AA: antioxidant activity; b*: color from blue to yellow; L*: luminosity; MG: microbiological growth; RR: respiration rate; SA: sensory attributes; SA: sensory attributes; and TA: titratable acidity.

## Data Availability

Not applicable.
